# Organic compounds in fluid inclusions of Archean quartz—Analogues of prebiotic chemistry on early Earth

**DOI:** 10.1371/journal.pone.0177570

**Published:** 2017-06-14

**Authors:** Ulrich Schreiber, Christian Mayer, Oliver J. Schmitz, Pia Rosendahl, Amela Bronja, Markus Greule, Frank Keppler, Ines Mulder, Tobias Sattler, Heinz F. Schöler

**Affiliations:** 1 Department of Geology, University Duisburg-Essen, Universitaetsstr. 5, Essen, Germany; 2 Faculty of Chemistry, CeNIDE, University Duisburg-Essen, Universitaetsstr. 5, Essen, Germany; 3 Faculty of Chemistry, Applied Analytical Chemistry, University Duisburg-Essen, Universitaetsstr. 5, Essen, Germany; 4 Institute of Earth Sciences, Ruprecht Karls University Heidelberg, Im Neuenheimer Feld 234–236, Heidelberg, Germany; 5 Heidelberg Center for the Environment (HCE), Ruprecht Karls University Heidelberg, Heidelberg, Germany; University of Saint Andrews, UNITED KINGDOM

## Abstract

The origin of life is still an unsolved mystery in science. Hypothetically, prebiotic chemistry and the formation of protocells may have evolved in the hydrothermal environment of tectonic fault zones in the upper continental crust, an environment where sensitive molecules are protected against degradation induced e.g. by UV radiation. The composition of fluid inclusions in minerals such as quartz crystals which have grown in this environment during the Archean period might provide important information about the first organic molecules formed by hydrothermal synthesis. Here we present evidence for organic compounds which were preserved in fluid inclusions of Archean quartz minerals from Western Australia. We found a variety of organic compounds such as alkanes, halocarbons, alcohols and aldehydes which unambiguously show that simple and even more complex prebiotic organic molecules have been formed by hydrothermal processes. Stable-isotope analysis confirms that the methane found in the inclusions has most likely been formed from abiotic sources by hydrothermal chemistry. Obviously, the liquid phase in the continental Archean crust provided an interesting choice of functional organic molecules. We conclude that organic substances such as these could have made an important contribution to prebiotic chemistry which might eventually have led to the formation of living cells.

## Introduction

One of the still unsolved mysteries in Science is the formation of prebiotic organic compounds on the prebiotic Earth. These organic compounds are prerequisite for the development of more complex molecular structures like lipids, proteins or nucleic acids, which were necessary components of the first living cell [1,2]. Several hypotheses are established on how organic compounds such as these have been formed and how they started to be integrated in processes leading to a living cell [3–8]. Alternatively and according to an earlier proposal, the first prebiotic chemistry and the formation of protocells could have occurred in the hydrothermal environment of tectonic fault zones in the upper continental crust, an environment where sensitive molecules are well protected against the destructive influence of UV radiation and could undergo complex reactions in a two-phase environment formed by hot water and supercritical carbon dioxide [9,10]. Tectonic fault zones offer a wide variety of reaction conditions regarding pressure, temperature, and catalytic surfaces. All reaction sites are interconnected by efficient material transport while isolated pockets allow for continuous reactions under constant conditions. Near a depth of 1 km (corresponding to T = 50°C and 100 bar of hydrostatic pressure), pressure variations caused by geysers or tidal phenomena cause a cyclic formation of vesicles [10]. At the same time, the whole system is under continuous supply of hydrothermal chemistry which is being formed at lower positions of the hydrothermal vents, maybe in a region between -2 and -4 km. Here, temperatures between 80° and 150°C as well as hydrostatic pressures between 200 and 400 bar provide the ideal reaction conditions for the formation of basic organic molecules.

**Fig 1:**
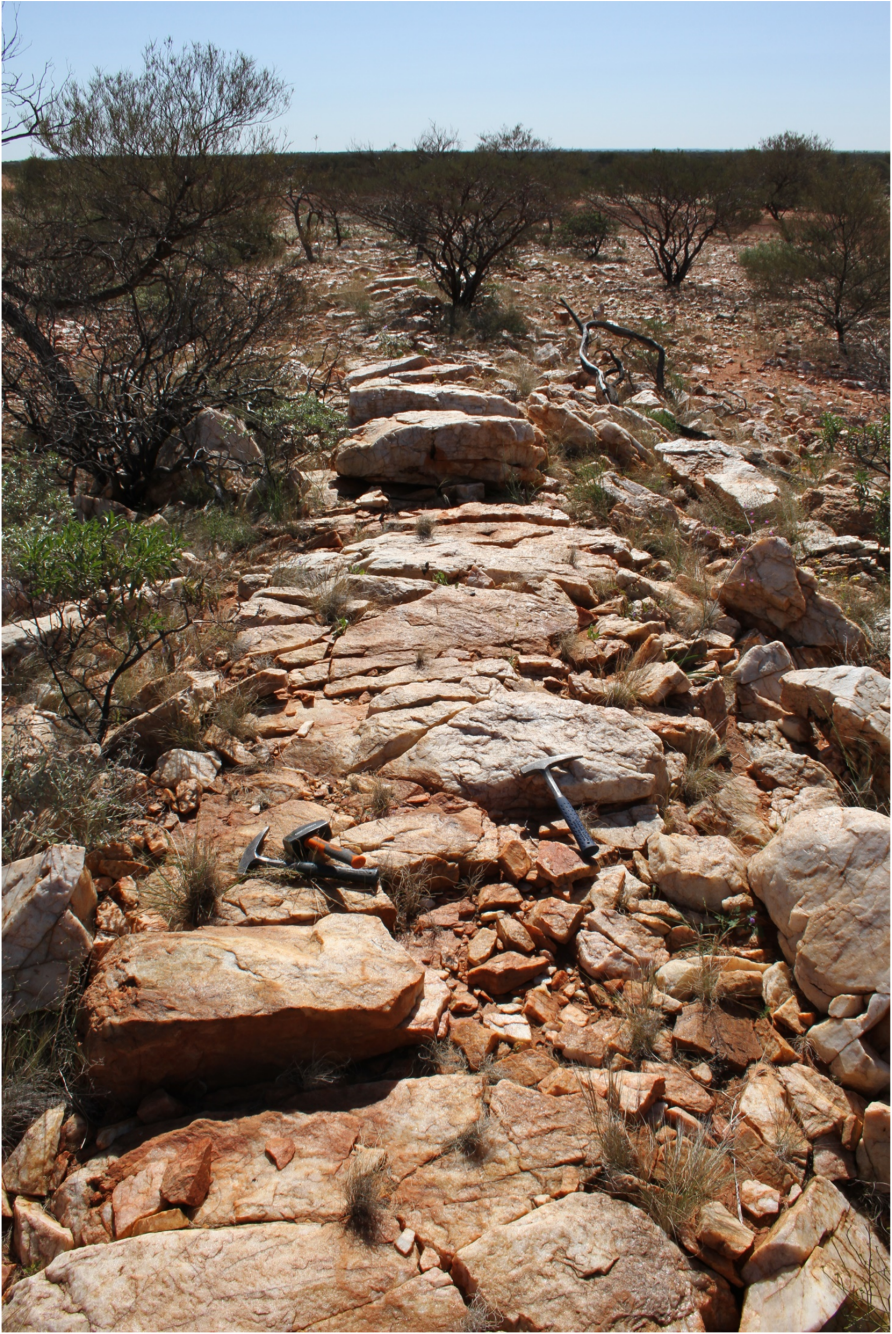
Remnant of a typical hydrothermal quartz dyke in the northern Jack Hills region / Western Australia which crystallized presumably in Archaean time in deeper parts of a shear-dominated crust (photograph by Thomas Kirnbauer, with permission).

Quartz crystals which have grown in this environment (an example of a hydrothermal quartz dyke is shown in Fig 1) should have entrapped small amounts of the fluid phase containing freshly formed organic compounds. Even though this fluid phase may have actually been formed at a point of time after the first living organisms already existed in other locations of the planet, it still would represent the original composition of the prebiotic stage provided that its origin is clearly abiotic.

In order to verify this hypothesis, a quartz dyke north of Jack Hills and >3 Ga old quartz pebbles from a conglomerate of the Jack Hills in Western Australia (a region where the oldest zircons, with an age of more than 4.3 Ga, were found [11–13]) were analyzed for organic constituents in their fluid inclusions (for details on the sites and on the sample collection see Text A in S1 File). Even though the ages of the quartz dyke and the fluid inclusions are poorly constrained and may well post-date the origin of life, this site represents an analogue for a setting that could plausibly have existed on the prebiotic Earth.

Initially, the crystals were carefully cleaned and analyzed for possible organic contaminants on their surface. In a second step, the material was further broken and ground to a fine powder. Finally, the organic components of the freshly released fluids were analyzed in detail by gas and liquid chromatography coupled with flame-ionization detector and mass spectrometry. An additional analysis using stable-isotope-ratio mass spectrometry was applied in order to prove the abiotic origin of the sample (see Text C in S1 File).

## Results

The content of organic substances in quartz crystals varied significantly from location to location. However, in contrast to minerals from the upper mantle (e.g. olivine from a mantle xenolith from a maar tuff ring, see Text H in S1 File), all quartz samples displayed at least minor concentrations of organic compounds in fluid inclusions. An example of the variety of molecular species found in quartz samples collected in and near Jack Hills is presented in Table 1.

**Table 1:** Organic compounds identified in hydrothermally grown quartz crystals near Jack Hills in Western Australia. The index letter “a” refers to the technical approach used for the analysis: a = 1 for one-dimensional gas chromatography with flame ionization detector^8^, a = 2 for comprehensive two-dimensional gas chromatography coupled to a quadrupole MS and a = 3 for liquid chromatography with a high-resolution TOF-MS.

compound class	compound	structure	a
Hydrocarbons	Methane	CH_4_	1
	Ethane	CH_3_-CH_3_	1
	Ethene	CH_2_ = CH_2_	1
	Ethyne	CH≡CH	1
	Propane	CH_3_-CH_2_-CH_3_	1
	Propene	CH_3_-CH = CH_2_	1
	n-Butane	CH_3_-CH_2_-CH_2_-CH_3_	1
	1-Butene	CH_3_-CH_2_-CH = CH_2_	1
	3-Methylpropene	(CH_3_)_2_CH = CH_2_	1
Halocarbons	2,2-Dichloroethanol	CHCl_2_-CH_2_OH	2
	1,1,2,2-Tetrachloroethane	CHCl_2_-CHCl_2_	2
	1,1,2,3-Tetrachloropropane	CHCl_2_-CHCl-CH_2_Cl	2
	3-Chloro-1-propanol	CH_2_Cl-CH_2_-CH_2_OH	2
	1,1-Dimethyl-3-chloropropanol	CH_2_Cl-CH_2_-C(CH_3_)_2_OH	2
	3,3-Dichloro-1-propanol	CHCl_2_-CH_2_-CH_2_OH	2
	1,3-Dichloro-2-propanone	CH_2_Cl-(CO)-CH_2_Cl	2
	2,2,3-Trichloropropional	CH_2_Cl-CCl_2_-CHO	2
	1,2,3-Trichloro-1-propene	CH_2_Cl-CCl = CHCl	2
Alcohols	4-Methyl-2-pentanol	CH_3_-CH(CH_3_)-CH_2_-CHOH-CH_3_	2
	3-Methyl-4-penten-1-ol	CH_2_ = CH-CH(CH_3_)-CH_2_-CH_2_OH	2
	3-Hexen-1-ol	CH_3_-CH_2_-CH = CH-CH_2_-CH_2_OH	2
	Tetramethyl-2-hexadecen-1-ol	(C_19_H_37_)-CH_2_OH	2
Aldehydes	Heptanal	C_6_H_13_-CHO	2
	Octanal	C_7_H_15_-CHO	2
	Nonanal	C_8_H_17_-CHO	2
	Decanal	C_9_H_19_-CHO	2
	Undecanal	C_10_H_21_-CHO	2
	Dodecanal	C_11_H_23_-CHO	2
	Tridecanal	C_12_H_25_-CHO	2
	Tetradecanal	C_13_H_27_-CHO	2
	Pentadecanal	C_14_H_29_-CHO	2
	Hexadecanal	C_15_H_31_-CHO	2
	Heptadecanal	C_16_H_33_-CHO	2
	3-Methoxybutyral	CH_3_-CH(OCH_3_)-CH_2_-CHO	2
Unknown		C_7_H_9_N_5_	3
		C_5_H_6_N_6_O_3_	3
		C_7_H_12_N_6_O_4_	3
		C_9_H_16_N_6_O_5_	3
		C_15_H_18_N_6_O_4_	3

Depending on the chemical composition, all compounds determined, except for those containing nitrogen, can be assigned to four classes: aliphatic hydrocarbons, halocarbons, alcohols, and aldehydes.

## Discussion

As a result of the given analytical method, the compounds listed under a = 1 and a = 2 reflect a fraction of substances which belongs to a given range of volatility. Therefore, the chlorinated molecules in Table 1 generally exhibit shorter chain lengths than the non-chlorinated compounds. Some of the aliphatic chains show methyl side groups, but most molecules exhibit unbranched backbones. The compounds listed under a = 3 could be determined only by their sum formula (see Text E in S1 File).

The collection of compounds contains some highly reactive species such as ethyne, alkenes, and aldehydes. Obviously, these compounds (or their precursors) must have been efficiently protected or stabilized in order to survive over billions of years. Given that the mineral environment inside the quartz inclusions offers a large choice of metal oxide surfaces (as from residues of transition metal cations which had originally been dissolved in the hydrothermal water), it is likely that the stabilization of some of these compounds occurred by formation of complexes with aldehydes or alkenes as single or multiple ligands [14, 15].

One may also speculate about the mechanisms of formation which have led to the given molecules. It is generally accepted that alkanes and alkenes may have been generated in analogy to the Fischer-Tropsch mechanism, catalyzed by various mineral surfaces present in the crustal cavities [16]. With chlorine or hydrogen chloride from volcanic sources, it is likely that the chlorinated compounds (which are actually known to occur in volcanic gases [17–20]) have formed as well. In fact, chlorine may have been the most efficient oxidizing agent under the given conditions. The monochlorinated alkanes and alkenes in turn are prone to hydrolysis in the presence of alkaline waters, hereby forming alcohols which are further oxidized to aldehydes, ketones, and carbonic acids [21]. Hence, the four classes of compounds listed in Table 1 could indicate a certain sequence of hydrothermal formation.

A very striking fact is the occurrence of the homologous series of aldehydes in the μg/kg concentration range (see Text G in S1 File). Within the given window of volatility, we were able to detect aldehydes with chain length between seven and seventeen carbon atoms (heptanal to heptadecanal) without any preference for even or uneven carbon numbers. The aldehydes were identified by spiking experiments with standard compounds (heptanal to hexadecanal). For heptadecanal, the retention time difference between the homologues and the mass spectra data bases were used for identification. These compounds have a strong potential to form amphiphilic units if the aldehydes are oxidized to fatty acids which (at higher pH values) form amphiphilic anions [22]. In addition, they may react with alcohols to form amphiphilic esters under hydrothermal conditions [22]. Therefore, they represent the chemical basis for larger associated structures like micelles, membranes, and vesicles which may have formed under the same conditions [4]. All the nitrogen-containing organic substances seem to be more polar and less volatile and could therefore be detected only by liquid chromatography (a = 3 in Table 1).

Even though the list of identified organic molecules does not contain known biomolecules, many substances observed can be considered their possible precursors: subsequent reaction with water and ammonia followed by an oxidation may turn most of the chlorinated compounds into amino acids [22]. As mentioned above, carboxylic acids which derive from the long-chained aldehydes may form various esters with the multivalent alcohols, leading to possible predecessors of lipid molecules [22]. The multivalent alcohols themselves may be seen as precursors of sugars [22].

**Fig 2:**
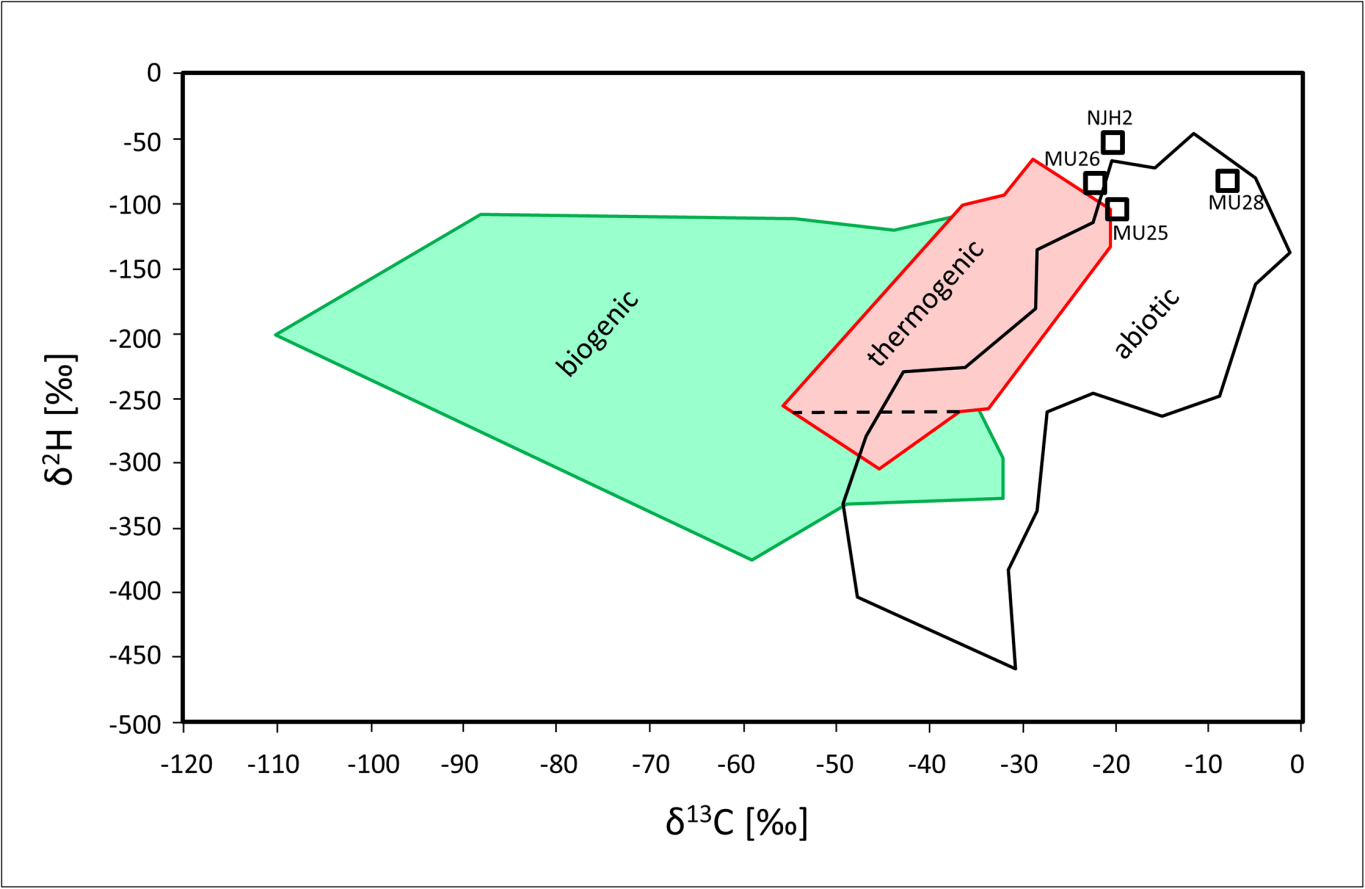
Stable carbon (δ^13^C) and hydrogen isotope (δ^2^H) composition of CH_4_ in the fluid inclusions of Australian quartz samples. For comparison, the plot shows data regions typical for biogenic sources (green), thermogenic sources (red) and abiotic sources (black) (modified after Etiope and Sherwood Lollar [23]). The codes assigned to the data points (black open squares) refer to the sample locations and are explained in Text A in S1 File.

Could other sources, such as contamination during the sample preparation or living organisms or thermal decomposition products thereof, have contributed to the observed organic substances? Our blanks clearly show that the sample preparation is not the source for the compounds listed in Table 1. Even though the composition of the identified organic compounds is atypical for petrochemistry, it is important to exclude that fossil organic material was the precursor for the organic compounds found in quartz samples. In order to support the hydrothermal origin of the enclosed contents, we measured the stable-isotope composition of CH_4_ in the samples that contained a variety of complex organic substances (see Text C in S1 File). The results of the stable carbon and hydrogen isotope measurements of CH_4_ are shown in Fig 2. The data regions generally assigned to biotic, thermogenic and abiotic methane sources according to Etiope and Sherwood Lollar [23] are indicated for comparison. The two-dimensional stable isotope results for CH_4_ clearly indicate that the CH_4_ found in the Australian quartz samples was formed from abiotic precursors such as carbon dioxide or elemental carbon and makes a biogenic (e.g. from microbes) or thermogenic origin (decomposition of organic matter) highly unlikely. Some data points are clearly found within and even beyond the region which has been assigned to abiotic methane on the basis of on present data. The data represent interesting counterparts to findings by Ueno et al. and Bell et al. who, by the same approach, could clearly prove that other fluid inclusions in hydrothermal precipitates from Pilbara Craton, Australia, and zirkons from Jack Hills, Australia, showed traces of methane and solid carbon deriving from biogenic origin [24, 25].

On this basis, it can be concluded that the organic compounds found in the inclusions have obviously been formed from abiotic sources by hydrothermal chemistry. Even though they may have been generated at a time where life was already established in other locations of the planet, they could be a possible example of the type of chemistry that may plausibly have occurred in similar settings on the prebiotic Earth. In fact, the chemical components listed in Table 1 have most likely been part of the fluid phase within the Earth’s crust from the very early beginning until the present day. On the primordial Earth, this or a similar mixture may have started the development and the fruitful evolution of prebiotic chemistry. In our opinion, these following steps may have occurred within the same environment, the system of hydrothermal vents, and may eventually have led to the formation of the first living cell.

## Materials and methods

### Collection of mineral samples from Jack Hills in Western Australia

Crystals from an undated quartz dyke north of Jack Hills (northern border of Yilgarn craton, Position: 25,875416° S, 117,242701° E) and pebbles from Archean conglomerates in the Jack Hills, Western Australia (Position: 26.167556° S, 116.990472° E) were collected. Permissions were not required for geological sampling in this given region as the land is publicly owned. The Yilgarn craton of Western Australia consists of granites, greenstones and granitic gneisses ranging from at least 3730 to 2550 Ma in age. A multitude of quartz dykes in NW, NE and EW directions have been exhumed during long lasting denudation of the Yilgarn craton. The Jack Hills, located in the Narryer Terrane of the northern Yilgarn Craton in Western Australia, comprise an approximately 80 km long northeast-trending belt of folded and weakly metamorphosed sedimentary rocks that include a mature clastic sedimentary rock series which has been interpreted as alluvial fan-delta deposits with an age of 2500–3000 Ma. In between these deposits, two conglomerates are intercalated.

### Stable isotope measurements of methane

Gas samples from the milling device [26] were injected directly into a continuous-flow isotope-ratio mass spectrometer (CF-IRMS) system for high-precision analysis of δ^13^C-CH_4_ and δ^2^D values [27]. δ^13^C (‰) values are reported relative to Vienna-PDB and defined by the equation δ^13^C = (R_sample_/R_V-PDB_-1) with R = ^13^C/^12^C. δ^13^C values (‰) are reported relative to Vienna Standard Mean Oceanic Water (VSMOW) and defined by the equation δ^2^H = (R_sample_/R_V-SMOW_-1) with R = ^2^H/^1^H.

For detailed information about the experimental setup and complete methods, we refer to the Text C in S1 File.

### Sample preparation

The rock samples were washed in concentrated sulfuric acid at 50°C for 30 min and afterwards washed with ultrapure water until the washing water was neutral. After the washing procedure, four Erlenmeyer flasks were used. The quartz samples were divided into three aliquots (each 12–13 g) in three Erlenmeyer flasks. The fourth Erlenmeyer flask was used as a blank (without sample material). Each sample was washed three times with 10 mL dichloromethane (DCM) for 10 min in an ultrasonic bath. The same procedure was performed with the empty Erlenmeyer flask (blank sample). After washing the surface of the quartz samples with DCM three times, the resulting DCM solutions of each sample including the blank were combined, concentrated and finally analyzed with comprehensive two-dimensional gas chromatography coupled to a quadrupole mass analyzer with the same parameter set as in the final analysis.

The washed quartz samples were milled with a SPEX 6850 Freezer Mill four times (each cycle at an amplitude 10 for 5 min). After milling, the quartz samples were immediately scraped out of the mill chamber with a spatula and collected in Teflon centrifuge tubes. Approximately 13 g of each milled sample was extracted with 10 mL DCM, centrifuged with a Beckman Coulter Allegra 25R Centrifuge for 5 min with 2000 rpm at 14°C, enriched to 150 or 1000 μL and finally analyzed with GCxGC-MS. Before grinding, the grinding devices (three pistons and vessels) were cleaned twice with water in an ultrasonic bath for 10 min to remove remaining grinding material. After drying, the pistons and vessels were rinsed three times with DCM and the third fraction of each of the three vessels was collected, evaporated first to 1000 μL. 450 μL of the 1000 μL volume was separated and evaporated to dryness, then re-dissolved in 150 μL DCM. Both samples were analyzed as additional blanks. Also a sample-free milling process was performed in order to exclude a source of contamination by the stainless steel containing carbon. For more detailed information please refer to the supporting information S1 File.

### Determination of organic compounds by GC-MS

GCxGC-MS was performed on a gas chromatograph equipped with a quadrupole mass analyzer (GC2010, GC-MS-QP2010 Ultra) from Shimadzu (Duisburg, Germany). Ionization was performed by electron impact (70 eV). Data were recorded and processed by GC-MS Solution software (Shimadzu, Duisburg, Germany) and GC Image (GC Image, LLC, Lincoln, Nebraska, USA). For detailed information about the chromatographic and mass spectrometric setup, we refer to the supporting information S1 File.

### Determination of organic compounds by LC-MS

An Agilent 1290 Infinity liquid-chromatography system was used, consisting of a 1290 Infinity binary pump (G4220A) with a Jet Weaver V35 mixer, a 1290 Infinity Flexible Cube Solvent Management module (G4227A), a 1290 Infinity HiP sampler (G4226A), a 1290 Infinity Thermostated Column compartment (G1316C) with an IM-qTOF-MS (Agilent 6560), equipped with a Dual Agilent Jet Stream electrospray ionization (AJS ESI) Source. The instrument was used in qTOF-only mode; that means the trapping gate is off and ions are just transferred through the drift tube to the rear funnel, so that there is no separation by ion mobility and the system is used like a conventional qTOF instrument. For detailed information about the chromatographic and mass spectrometric setup, we refer to the supporting information S1 File.

## Supporting information

S1 File (PDF)
