# Supplementary material for: Organic compounds in fluid inclusions of Archean quartz—Analogues of prebiotic chemistry on early Earth
Source: PLoS One. 2017 Jun 14;12(6):e0177570. doi: 10.1371/journal.pone.0177570 (PMC5470662; doi:10.1371/journal.pone.0177570)
Supplement: S1 File — (PDF) [file pone.0177570.s001.pdf]

Supplementary Material

Organic compounds in fluid inclusions of Archean quartz - analogues of prebiotic chemistry on early Earth

Ulrich Schreiber[1], Christian Mayer[2], Oliver J. Schmitz[3], Pia Rosendahl[3], Amela Bronja[3], Markus Greule[4], Frank Keppler[4,5], Ines Mulder[4], Tobias Sattler[4], Heinz Friedrich Schöler[4,5]

[1]Department of Geology, University Duisburg-Essen, Universitaetsstr. 5, 45117 Essen, Germany

[2]Faculty of Chemistry, CeNIDE, University Duisburg-Essen, Universitaetsstr. 5, 45117 Essen, Germany

[3]Faculty of Chemistry, Applied Analytical Chemistry, University Duisburg-Essen, Universitaetsstr. 5, 45117 Essen, Germany

[4]Institute of Earth Sciences, Ruprecht Karls University Heidelberg, Im Neuenheimer Feld 234-236, D-69120 Heidelberg, Germany

[5]Heidelberg Center for the Environment (HCE), Ruprecht Karls University Heidelberg, D-69120 Heidelberg, Germany

* Corresponding author

E-Mail: christian.mayer@uni-due.de

Content

A Sample locations 2

B Petrographic characteristics of the quartz dyke sample 3

C Stable-isotope analysis of methane 5

D Analysis of volatile organic compounds in the liquid inclusions 6

E Analysis of semi-volatile compounds in the liquid inclusions 7

F Analysis of more polar compounds with LC-MS........ 14

G Semi-quantitative analysis of aldehydes in liquid inclusions 15

H Analysis of an olivine sample with GCxGC-MS 17

I References........ 18

A Sample locations

Crystals from an undated quartz dyke north of Jack Hills (northern border of Yilgarn craton) and pebbles from Archean conglomerates in the Jack Hills, Western Australia were collected and analyzed for possible residues of the ancient organic chemistry. As a reference specimen an olivine-rich mantle xenolith from the Quaternary Western Eifel, Germany, volcanic field has been used.

The Yilgarn craton of Western Australia consists of granites, greenstones and granitic gneisses ranging from at least 3730 to 2550 Ma in age. The main tectonic, volcanic, plutonic, and metamorphic activity occurred between 2780 and 2630 Ma ago in a major episode of plate tectonic activity which at least formed the Yilgarn craton. Subsequently, the craton underwent a number of tectonic impacts in which faults with hydrothermal mineralization might develop. The structural framework in the northeastern Yilgarn craton, for example, was largely shaped by transpression (2655 Ma – 2635 Ma ago) that led to the development of folds, reverse faults and sinistral strike-slip movements on NNW-trending regional shear zones[1]. A multitude of quartz dykes in NW, NE and EW directions have been exhumed during long lasting denudation of the Yilgarn craton[2] (2 to 4 km since 250 Ma). Most of them show hydrothermal origin as documented by open spaces with euhedral quartz crystals. The sampled quartz dyke north of Jack Hills (Fig S-1) is significantly younger than the highly metamorphic host rock and was therefore not affected by the high grade metamorphism of the host rock. Due to the lack of specific metamorphic minerals in this dyke a reasonable estimation of the p/T path cannot be made; quartz minerals are stable over a wide range of conditions. From the estimated denudation of up to 4 km since the last 250 Ma and the minimum age of the last orogenic impact the p/T range (based on 4 km depth) can be assumed of 50 -100 MPa and 80 – 160 °C (with 40 °C/km geothermal gradient, Drury S.A. Nature 1978, Vol 274, p 720-721).

The Jack Hills, located in the Narryer Terrane of the northern Yilgarn Craton in Western Australia, comprise an approximately 80 km long northeast-trending belt of folded and weakly metamorphosed sedimentary rocks that include a mature clastic sedimentary rock series which has been interpreted as alluvial fan-delta deposits with an age of 2500 – 3000 Ma[3]. Here the oldest zircons with an age of approximately 4300 Ma were found[3,4,5]. In between these deposits, two conglomerates are intercalated. It is assumed that some quartz pebbles of these conglomerates are from hydrothermal dykes. About the history of the hydrothermal quartz pebbles, it can be said that they have to be significantly older than the deposit.

The metamorphic history of the Jack Hills is not well known (Cavosie et al. 2004). Baxter et al. (1984) and Elias (1983) suggest from some indicator minerals that the siliclastic rocks experienced greenschist to lower amphibolite facies metamorphism. But no modern investigation about the p/T path exists. Furthermore, the strength (hardness) of the unweathered siliclastic rocks is lower than quartzites from amphibolite facies type. Sedimentary structures, for example the cross bedding, are well preserved. So we assume that the grade of metamorphism reaches only the lower greenschist facies with temperatures in the range of the analytical method.

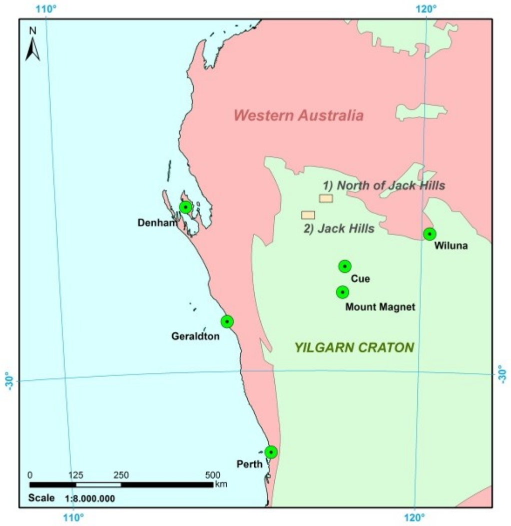

Figure 1 Map of Western Australia with Jack Hills sampling sites

1) Position: 25,875416° S, 117,242701° E: Quartz dyke in 2,6 By biotite-monzogranite[6]

2) Position: 26.167556° S, 116.990472° E: Quartz pebbles in conglomerates[7]

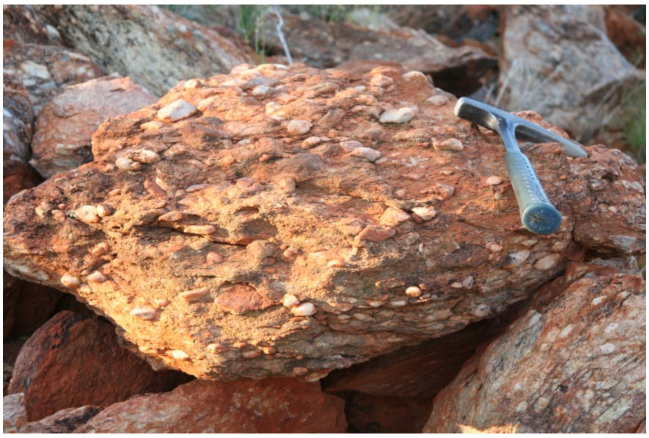

Figure S-2 Quartz pebbles in conglomerate from location (2), Jack Hills

B Petrographic characteristics of the quartz dyke sample

The sample NJH2 is collected from a quartz dyke north of the Jack-Hill Mountains. The dyke is exposed in a 2,6 By old granitic host rock[6] and is mainly composed of fine- grained cataclastic quartz (less than 500 µm) with different numbers of secondary hydrothermal dykes (diameters from millimeter to decimeter). As an example, the photomicrographs in Figure S-3 show a typical hydrothermal quartz dyke (more than 2 cm in diameter) surrounded by fine-grained quartz. Quartz crystals are grown nearly symmetrical from the boundary toward the center of the dyke. Fluid inclusions (small black dots) distributed along the crystal faces show growth zones of the host quartz. The textures indicate that these infiltrations represent primary inclusions[8,9]. A second generation of inclusions is distributed in parallel planes. Some of them intersect between different quartz crystals. Almost all inclusions are distinctly smaller than the minimum size which is necessary for further analyses like Raman spectroscopy.

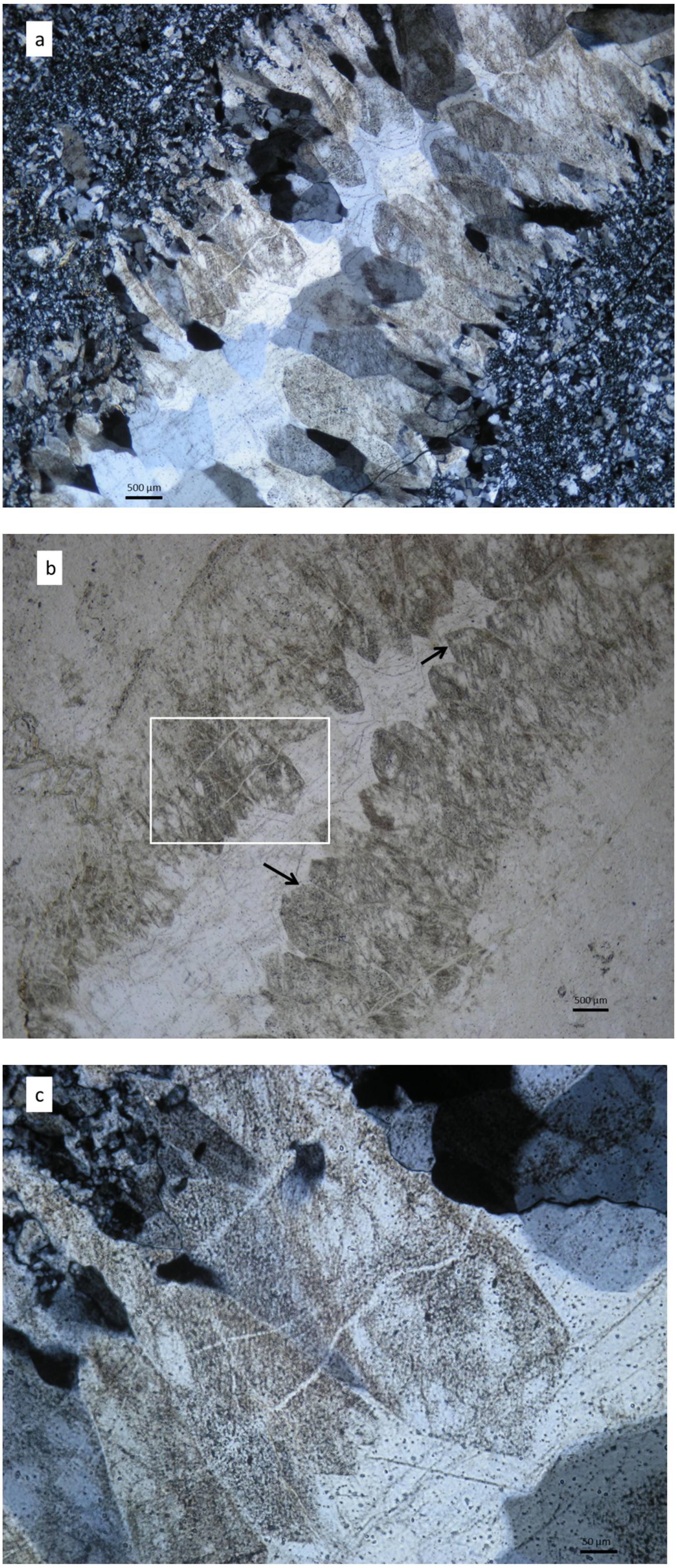
a
500 µm
b
500 µm
c
50 µm

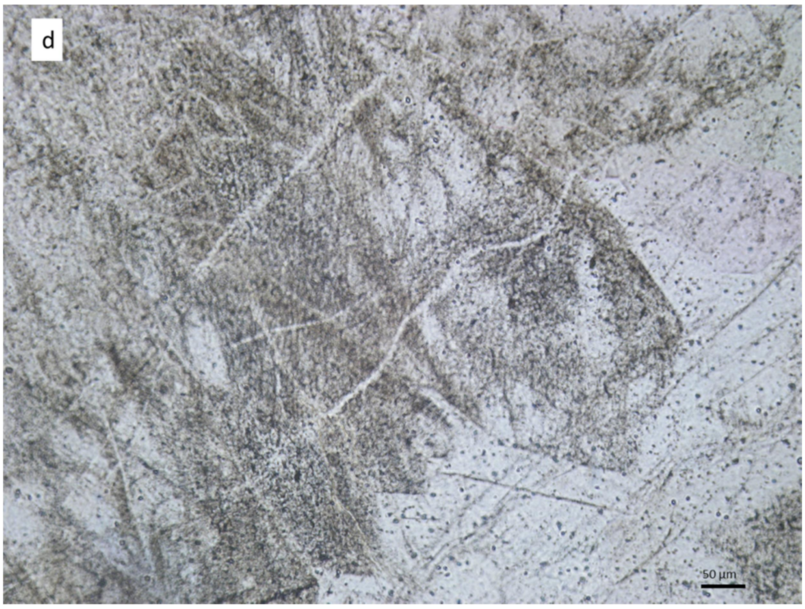

Figure S-3 **a**) Photomicrograph (obtained under cross-polarized light) of fluid inclusions in a typical quartz dyke (ca. 2 mm in diameter) north of Jack-Hill Mountains **b**) Photomicrograph (obtained under plane-polarized light) of the same section as **a**). Fluid inclusions (small black dots) distributed along the crystal faces show growth zones of the host quartz (arrows). **c**) Enlarged view (obtained under cross-polarized light) of **a**) corresponding to the white box in **b**. **d**) Enlarged view of **b**) (obtained under plane-polarized light). A second generation of inclusions is distributed in parallel planes. Some of them intersect two different quartz crystals (lower right section of the photomicrograph).

Conglomerate

Due to the small sizes of conglomeratic pebbles, the entire substance has been consumed for analysis of the organic chemistry. Additional petrographic investigations have not been possible.

Reference specimen:

Mantle xenolith (Lherzolite) from the tuff wall of Meerfelder Maar Quaternary West Eifel volcanic field10[10] (50.099882° N; 6.739577° E). The xenolith with a diameter of nearly 8 cm was in contact with subsurface water during the last 30.000 years.

C Stable-isotope analysis of methane

C1 Stable-carbon-isotope analysis of CH_4

Stable-carbon-isotope ratios ($^{13}C/^{12}C$) of CH_4 were analyzed with an in-house-built cryogenic preconcentration unit coupled to a gas chromatography-combustion-isotope ratio mass spectrometer (GC-C-IRMS). Aliquots of the gas samples with CH_4 mixing ratios of 1.8 to 2.5 ppmv were transferred to an evacuated 40 ml sample loop. CH_4 was trapped on Hayesep D at -125°C, separated from interfering compounds by gas chromatography and transferred via an open split to the isotope-ratio mass spectrometer (Thermo Finnigan Delta^{plus} XL, Bremen, Gemany). All $^{13}C/^{12}C$ -isotope ratios are expressed in the conventional δ notation in per mil versus V-PDB, [‰] versus VPDB with the use of the following equation 1:

$\delta^{13}C_{V\text{-}PDB} = ((^{13}C/^{12}C_{sample}) / (^{13}C/^{12}C_{standard})) - 1.$ Equation 1

A tank of high-purity CO_2 (carbon dioxide 4.5, Messer Griesheim, Frankfurt, Germany) with a calibrated $\delta^{13}C$ value of -23.63‰ (VPDB) was used as a working reference gas (calibrated at MPI for Biogeochemistry, Jena, Germany). All $\delta^{13}C$ values obtained from analysis of CH_4 were corrected by use of three CH_4 working standards (isometric instruments, Victoria, Canada) calibrated against IAEA and NIST reference substances. The calibrated $\delta^{13}C$ values of the three working standards in ‰ vs. V-PDB were -23.9±0.2‰, -38.3±0.2‰ and -54.5±0.2‰.

C2 Stable-hydrogen isotope analysis of CH_4

Stable-hydrogen isotope ratios ($^2H/^1H$) of CH_4 were analyzed with the same system as described for $\delta^{13}C$ measurements. All $^2H/^1H$-isotope ratios are expressed in the conventional δ notation in per mil versus V-PDB, [‰] versus VPDB with the following equation 2:

$\delta^2H_{V\text{-}SMOW} = ((^2H/^1H_{sample}) / (^2H/^1H_{standard})) - 1.$ Equation 2

A tank of high-purity H_2 (Alphagaz 2, (hydrogen 6.0), Air Liquide, Düsseldorf, Germany) in the range of -250‰ (not calibrated) was used as a working reference gas. All δ^2H values obtained from analysis of CH_4 were corrected on the basis of two CH_4 working standards (isometric instruments, Victoria, Canada) calibrated against IAEA and NIST reference substances. The calibrated δ^2H values of the working standard in ‰ vs. V-SMOW were -144±4‰ and -138±4‰.

	δ^2H [‰]	deviation	$\delta^{13}C$ [‰]	deviation	
**MU25**	-103.57		-19.472		
**MU26**	-83.36	4.55	-21.992		
**MU28**	-79.09	48.39	-7.607	2.250	
**NJH2**	-37.00		-6.267		
values without deviation are individual measurements					

Table S-1 Stable-isotope analysis of methane in the liquid inclusions

D Analysis of volatile organic compounds in liquid inclusions

Quartz crystals were pre-crushed to 3 mm grain sizes with a jaw crusher. Afterwards crystals were washed several times in ethanol in an ultrasonic bath to eliminate surface contaminations. For elimination of surface adsorbed gases, quartz crystals were evacuated to < 10^{-3} mbar. Quartz samples (10 g) were ground for 10 min at 400 rpm in a grinding device designed by Mulder et al. 2013[11] for a planetary mill (Fritsch Pulverisette 5, Idar Oberstein, Germany).

The released gases were subsequently analyzed with a gas chromatograph (Varian Star 3400 CX, Walnut Creek, California, USA) and an ion-trap mass spectrometer (Varian Saturn 2000, Walnut Creek, California, USA). The capillary columns employed in the GC were a DB-624, internal diameter 0.32 mm, film thickness 1.8 µm, 30 m, directly connected to a BP-5, internal diameter 0.32 mm, film thickness 1 µm, 60 m column. For more details on the dynamic injection and pre-concentration by the customized purge-and-trap system as well as the grinding procedure, please refer to Mulder et

al. 2013[11]. Short-chained hydrocarbons were determined with a gas chromatograph (Vega 6000, Carlo Erba Strumentazione, Italy) coupled to a flame ionization detector (FID). The column employed in the GC was a J&W HP-Al/S, internal diameter 0.53 mm, film thickness 15 µm, 30 m.

E Analysis of semi-volatile compounds in the liquid inclusions

E1 Sample Preparation

The stones were washed in concentrated sulphuric acid at 50°C for 30 min (Figure S-3) and afterwards washed with ultrapure water until the wash water was neutral.

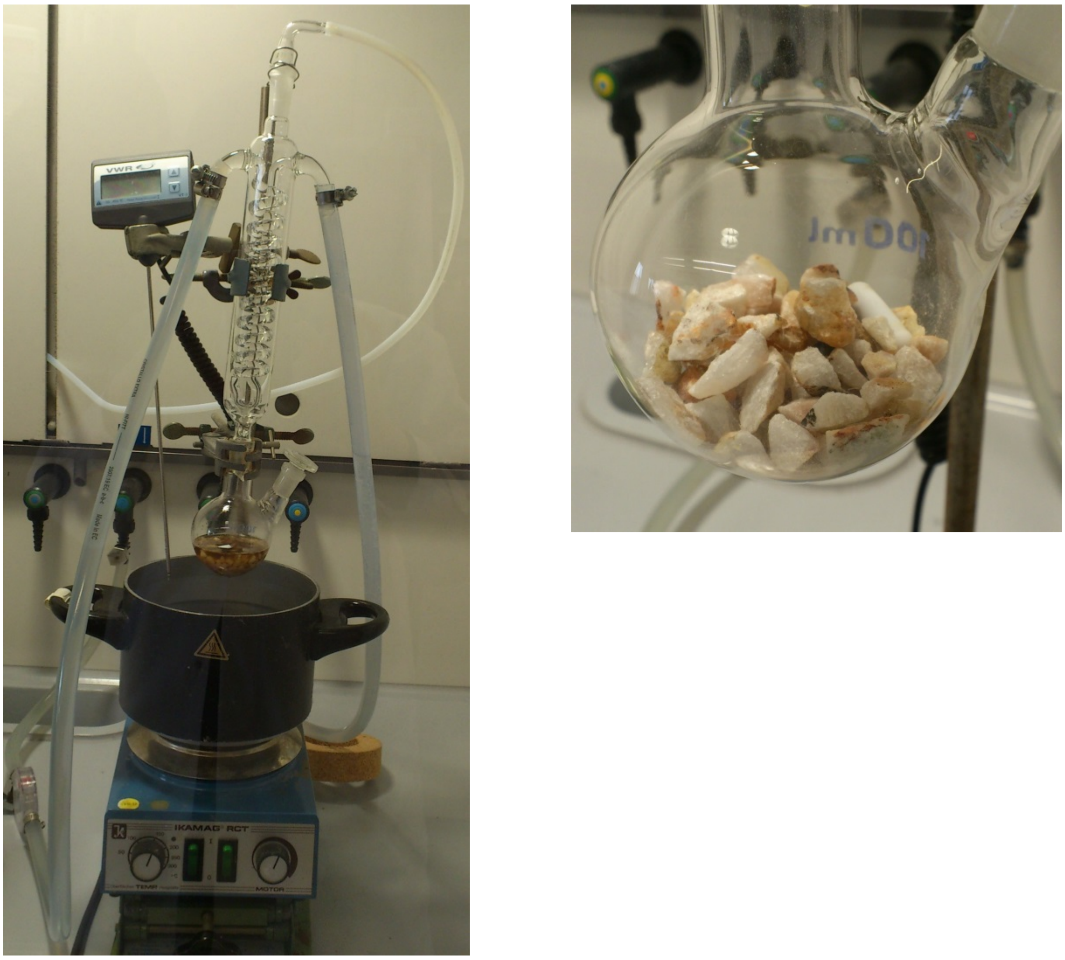

Figure S-4 H_2SO_4 pretreatment equipment

After the washing procedure, four Erlenmeyer flasks were used. The quartz samples were divided into three aliquots (each 12-13 g) in three Erlenmeyer flasks. The fourth Erlenmeyer flask was used as a blank (without samples). Each sample was washed three times with 10 mL dichloromethane (DCM) for 10 min in an ultrasonic bath. The same procedure was performed with the Erlenmeyer flask for the blank.

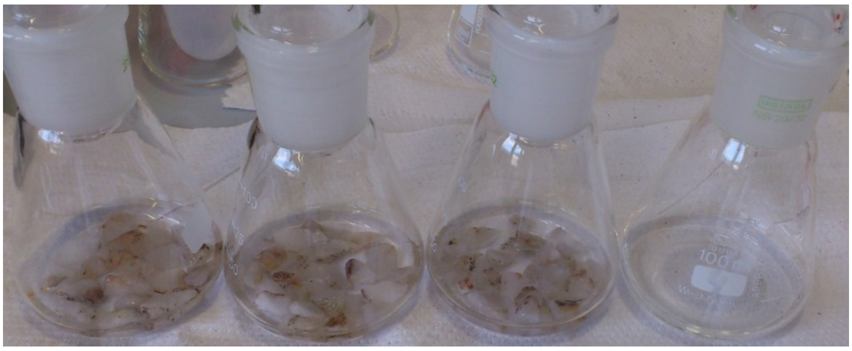

Figure S-5 Washed quartz samples in Erlenmeyer flasks

After washing the surface of the quartz samples with DCM three times, the resulting DCM solutions of each sample including the blank were combined, concentrated and finally analyzed with comprehensive two-dimensional gas chromatography coupled to a quadrupole mass analyzer with the same parameter set as in the final analysis.

The enrichment of the wash solutions was done with Büchi Syncore Q101 at 30°C, 250 rpm and 400 mbar to a final volume of 1000 µL (Figure S-5). These samples were used to analyze very volatile compounds such as chlorinated hydrocarbons. To obtain better MS-spectra for the compounds with a lower vapour pressure, the samples were further enriched with 450 µL of the 1000 µL volume and evaporated to dryness with the same Büchi Syncore at 35°C, 260 rpm and 400 mbar. The analytes were then dissolved in 150 µL DCM. During this process most of the low volatiles were lost.

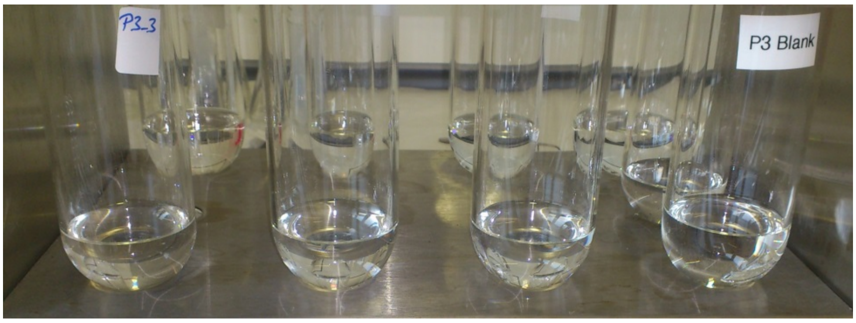

Figure S-6 Extracts before evaporation in the Büchi Syncore

As an example, the three GCxGC-MS analyses of the DCM-extracts of control samples NJH2 (a, b, c) and the corresponding blank (d, e) are shown in Figure S-6 (a-e):

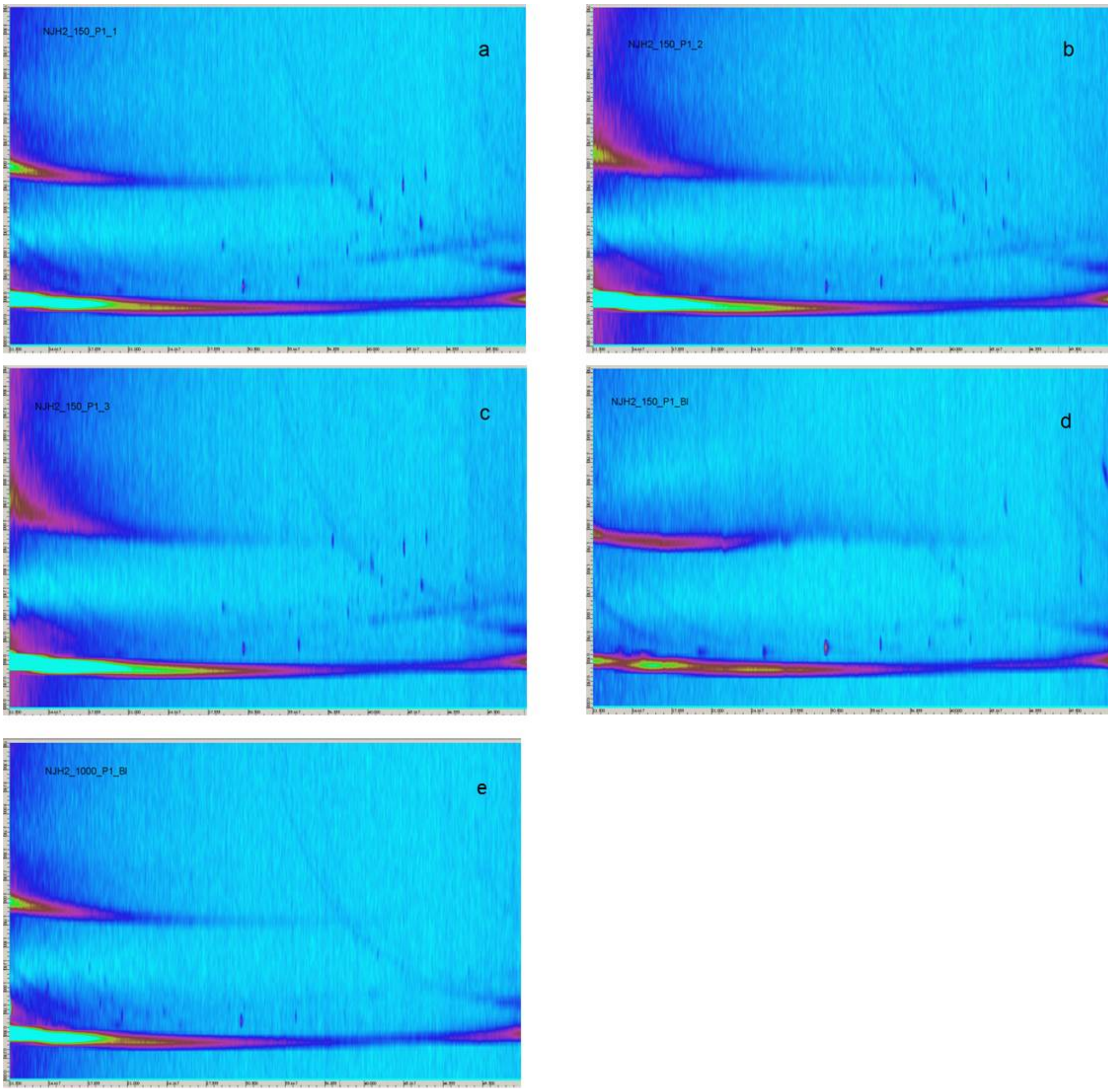

Figure S-7 GCxGC-MS analysis of control samples from sample NJH2 (a, b, c) after enrichment to 150 µL and the corresponding blank d (enrichment to 150 µL) and e (enrichment to 1000 µL).

The washed quartz samples were milled with a SPEX 6850 Freezer Mill four times (each cycle at amplitude 10 for 5 min). After milling the quartz samples were immediately scraped out of the mill chamber with a spatula and collected in Teflon centrifuge tubes.

Before grinding, the grinding devices (three pistons and vessels) were cleaned twice with water in an ultrasonic bath for 10 min to remove previous grinding material. After drying, the pistons and vessels were rinsed three times with DCM and the third fraction of each of the three vessels was collected and evaporated first to 1000 µL with the Büchi syncore. 450 µL of the 1000 µL volume was used and evaporated to dryness, then dissolved in 150 µL DCM. Both were analyzed as additional blanks. Approximately 13 g of each milled sample was extracted with 10 mL DCM, centrifuged with a Beckman

Coulter Allegra 25R Centrifuge for 5 min with 2000 rpm at 14°C, enriched to 150 or 1000 µL (the parameter set was the same as described before) and finally analyzed with GCxGC-MS.

E2 GCxGC-qMS analysis

As an example, the three GCxGC-MS analyses of the sample NJH2 (a, b, c) and the corresponding blank (d, e) are shown in Figure S-7:

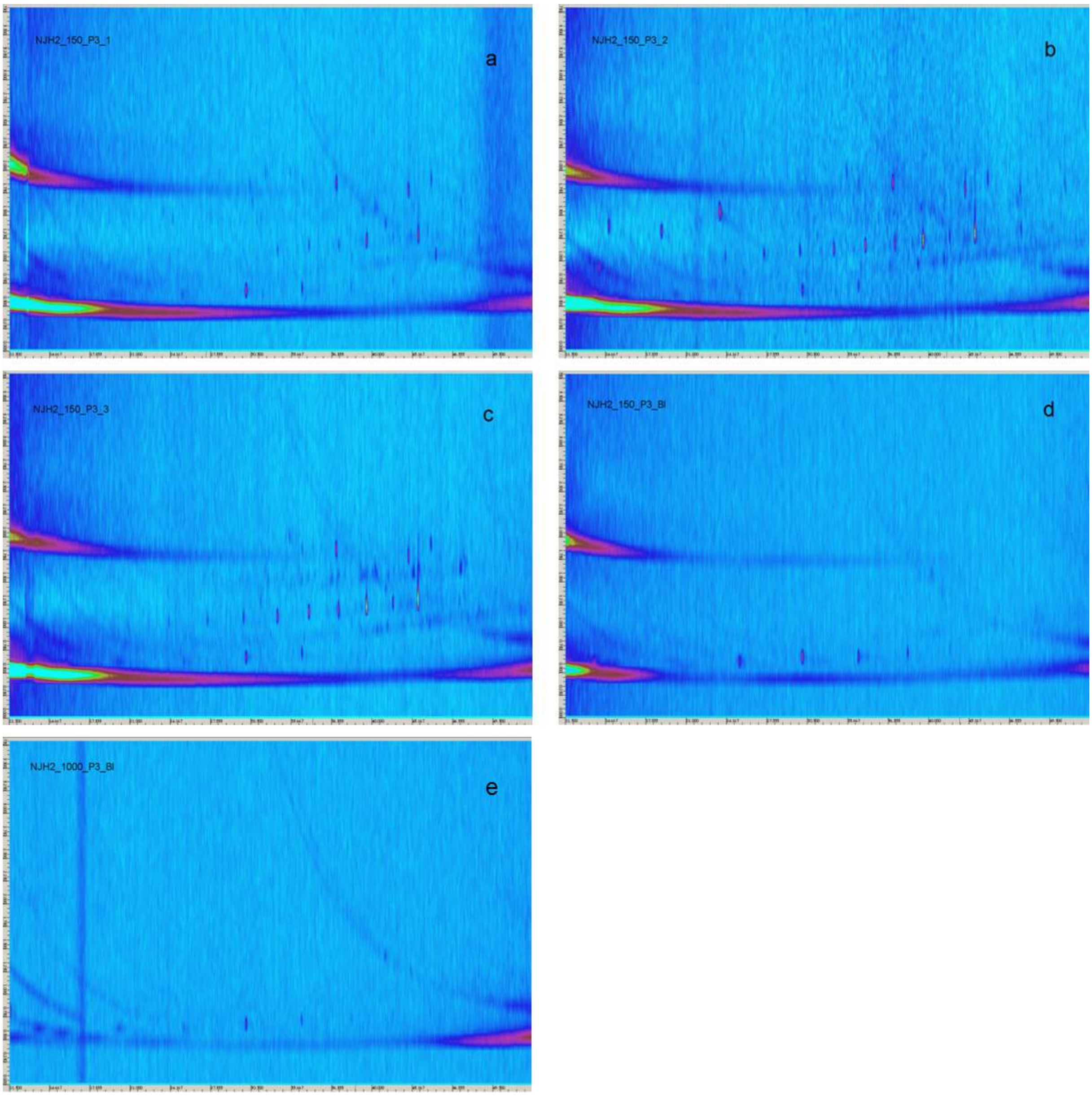

Figure S-8 GCxGC-MS analyses of sample NJH2 (a, b, c) after enrichment to 150 µL and the corresponding blank d (enrichment to 150 µL) and e (enrichment to 1000 µL)

As an example, the GCxGC-MS analysis of one aliquot of NJH2 is shown in more detail in Figure S-8, and the results are listed in Table S-2.

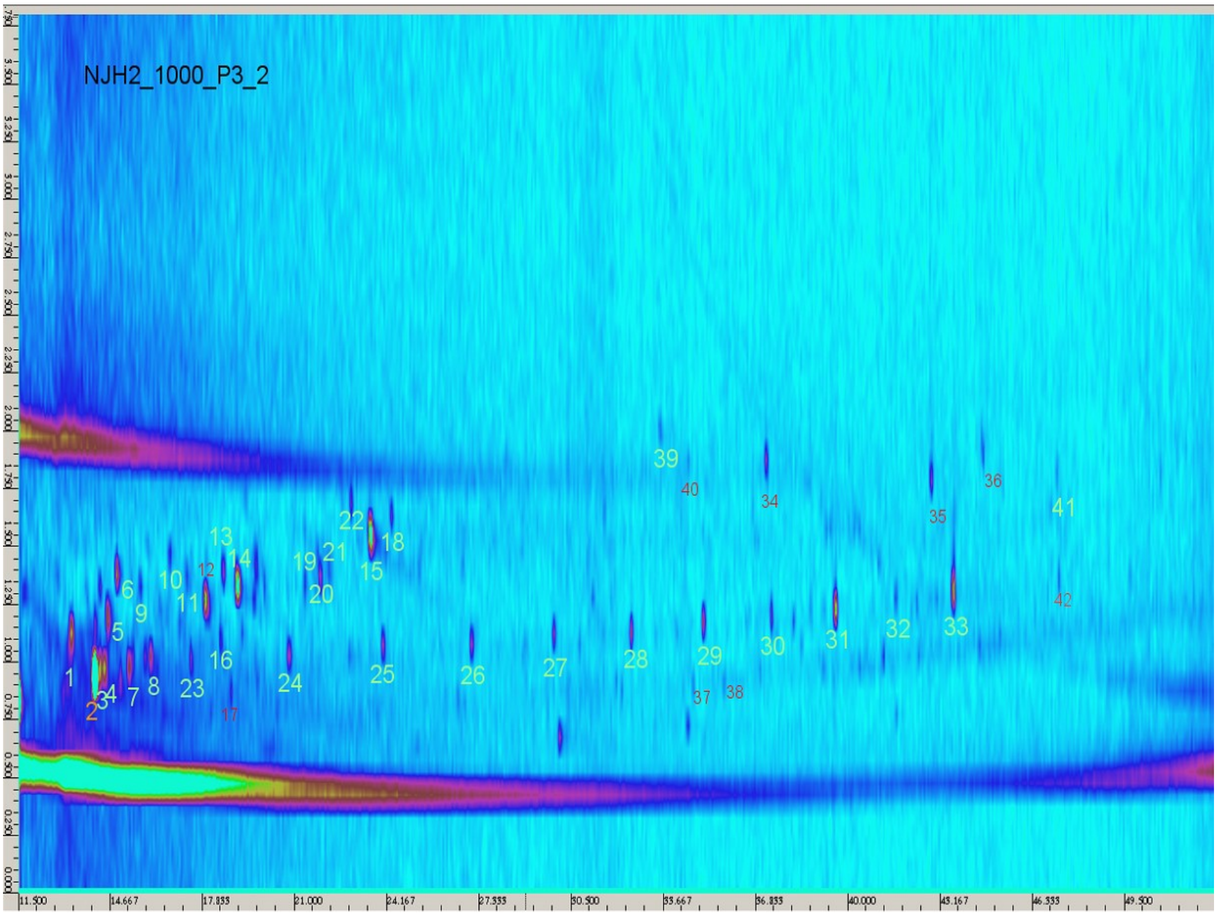

Figure S-9 GCxGC-MS analysis of one aliquot of NJH2 after enrichment to 1000 µL

Table S-2 GCxGC-MS analysis of one aliquot of NJH2 after enrichment to 1000 µL with the suggestion of the libraries NIST and W9N08, the retention times in the first and second dimension (RT_{1D} and RT_{2D}) and the library matches.

Number	Suggestion library (NIST and W9N08)	RT_{1D} [min]	RT_{2D} [s]	Match [%]
1	2,2-Dichloroethanol	13.273	1.10	85
2	3-Hexen-1-ol, (E)	14.097	0.90	92
3	1,1-Dimethyl-3-chloropropanol	14.287	0.95	82
4	4-Methyl-2-pentanol	14.477	0.95	90
5	3-Chloro-1-propanol	14.540	1.15	93
6	2,3-Dichloro-1-propanol	14.857	1.35	75
7	3-Methyl-4-penten-1-ol<br>4-Hexen-1-ol	15.300	0.95	90<br>88
8	3-Hexen-1-ol, (Z)<br>3-Methyl-1-penten-3-ol	16.06	1.00	88<br>88
9	1,2,3-Trithiolane	15.617	1.30	67

Number	Suggestion library (NIST and W9N08)	RT_{1D} [min]	RT_{2D} [s]	Match [%]
10	1,3-Dichloro-2-propanone	16.630	1.45	85
11	1,3-Dichloro-2-propanol<br>3-Chloro-2-hydroxypropanoic acid	17.263	1.35	77<br>76
12	1,1,2,2-Tetrachloroethane	17.897	1.25	95
13	Chloroacetyl chloride	18.530	1.35	73
14	3,3-Dichloropropan-1-ol	18.973	1.35	84
15	2,2,3-Trichloropropional<br>2,2,3-Trichloropropan-1,1-diol	23.533	1.50	82<br>81
16	3-Methoxybutyral<br>2-Methyl-3-pentanol	18.403	1.05	81<br>81
17	α-Pinene	18.783	0.83	82
18	3-Pyridinesulphonic acid	24.230	1.60	54
19	2,2-Dichlorobutane	21.317	1.30	63
20	1,1,2,3-Tetrachloropropane	21.823	1.35	89
21	1,4-Dichlorobutene	22.140	1.35	71
22	1,2,3-Trichloro-1-propene	22.900	1.65	90
23	Heptanal	17.390	0.95	87
24	Octanal	20.747	1.00	91
25	Nonanal	24.040	1.05	91
26	Decanal	27.017	1.05	92
27	Undecanal	29.867	1.10	91
28	Dodecanal	32.527	1.10	91
29	Tridecanal	34.997	1.15	92
30	Tetradecanal	37.340	1.20	92
31	Pentadecanal	39.557	1.20	93
32	Hexadecanal	41.583	1.25	90
33	Heptadecanal<br>Hexadecanal<br>Pentadecanal	43.610	1.30	---<br>92<br>92
34	Diethyl phthalate	37.150	1.85	90
35	Isobutyl phthalate	42.850	1.75	90
36	Dibutyl phthalate	44.623	1.90	79
37	3,7-Dimethylnonane	34.617	0.85	89
38	4,5-Dimethylnonane	35.693	0.90	74
39	1,3-Diacetylbenzene	33.477	2.00	72
40	2,6-Diacetylpyridine	34.490	1.85	68
41	2,2'-[1,10-<br>Decanediylbis(oxy)]bis(tetrahydro-2H-	47.157	1.80	70

Number	Suggestion library (NIST and W9N08)	RT_{1D} [min]	RT_{2D} [s]	Match [%]
	pyran)			
42	Methyl n-tetradecanoate	47.220	1.35	78

Brown: This compound was also detected in the extracted unmilled quartz sample but at much lower intensity.
Red: These compounds were also detected in one of the blank samples.

GCxGC-MS was performed on a gas chromatograph equipped with a quadrupole mass analyzer (GC2010, GC-MS-QP2010 Ultra) from Shimadzu (Duisburg, Germany). Ionization was performed by electron impact (70 eV). Data were recorded and processed by GC-MS Solution software (Shimadzu, Duisburg, Germany) and GC Image (GC Image, LLC, Lincoln, Nebraska, USA). The carrier gas was helium (flow: 1.96 mL/min), and a ZX1-LN2 cooled loop modulator from Zoex was used as a cryo-interface (Zoex Corporation, Houston, Texas, USA). The effluent from the non-polar column in the first dimension (ZB-5MSi 30m, 0.25 mm i.d., 0.25 µm df) from Phenomenex (Aschaffenburg, Germany) was modulated (modulation frequency 3.8 s, Hot-Jet: 200 ms, 130°C, after 10 min 280°C) and separated on a moderately polar second-dimension column (ZB-50, 1.7 m, 0.1 mm i.d., 0.1 µm df) from Phenomenex (Aschaffenburg, Germany). The injection volume was 2 µL (split ratio: 1:10), and the injector temperature was 310°C. The oven temperature started at 40°C (hold 1 min) and was then ramped at 5 °/min to 300°C (hold 1 min).

The ionization- and MS-parameters are listed in Table S-3:

Table S-3 Ionisation- and MS-parameter

**Ionisation- and MS-Parameter**	
Electron Ionisation	70 eV
Ion source temperature	200°C
Interface temperature	310°C
Solvent Cut Time	11.00 min
MS-Start Time	11.50 min
MS-End Time	53.00 min
ACQ Mode	Scan
Event Time	0.05 s
Scan Speed	20,000
m/z-range	40-850

The following table lists the signal-to-blank ratios for a choice of compounds from table S-2:

Table S-4 Signal-to-blank ratios for selected compounds from Table S-2

		S/N
Halogenated Compounds		
1	2,2-Dichloroethanol	3.3
14	3,3-Dichloropropan-1-ol	9.6
12	Ethane, 1,1,2,2-tetrachloro-	6.4
15	2,2,3-Trichloropropionaldehyde	14.4
Alcohol		
4	4-Methyl-2-pentanol	2.8
Aldehydes		
31	Pentadecanal	25.8
33	Heptadecanal	22.5

F Analysis of more polar compounds with LC-MS

The quartz sample NJH2 was treated almost the same as in the gas chromatographic sample preparation, but with methanol as solvent. The only difference was in the subsequent treatment of the collected extracts. The enrichment of the samples was done with Büchi Syncore Q101 at 45°C, 250 rpm and 200 mbar to a volume of 1000 µL. For the final measurement, 200 µL of the enriched extract were taken and added with 200 µL ultrapure water. 10 µL were injected into an HPLC system. An Agilent 1290 Infinity liquid-chromatography system was used, consisting of a 1290 Infinity binary pump (G4220A) with a Jet Weaver V35 mixer, a 1290 Infinity Flexible cube solvent management module (G4227A), a 1290 Infinity HiP sampler (G4226A), a 1290 Infinity Thermostated Column compartment (G1316C) with an IM-qTOF-MS (Agilent 6560), equipped with a Dual Agilent Jet Stream electrospray ionization (AJS ESI) Source. The instrument was used in qTOF only mode; that means the trapping gate is off and ions are just transferred through the drift tube to the rear funnel so that no ion mobility separation occurs and the system is used like a conventional qTOF instrument. For the separation, an Agilent Zorbax Eclipse Plus C18 column (3.5 µm, 100 mm x 4.6 mm) was used. The flow rate was 400 µL/min, and the binary mobile phase consisted of (A) water (0.1% FA) and (B) methanol (0.1% FA). The LC run time was 40 min with the following gradient profile: 0-5 min, 95% A and 5% B; 5-35 min linear gradient to 5% A and 95% B and maintained from 35-40 min. The ionization was in ESI positive mode with a gas temperature of 200°C; the gas flow was 3 L/min, the nebulizer pressure 20 psig, the sheath gas temperature 325°C and the sheath gas flow rate 12 L/min.

Data evaluation:

Three positive samples (aliquots of NJH2) and the additional 6 control samples (blanks) were analyzed. After feature analysis of these resulting high resolution LC-MS data with the software Agilent Mass Hunter Profinder B.06.00, all features which could be determined in three samples were listed. After that, we checked which features belong only to the three mill charge samples. The *m/z* values of these features were noted, and formulas were generated with the software Agilent Mass Hunter Qualitative B.07.00. For each *m/z* the formula with the highest score was selected. That means that we have only identified compounds which were in each of the three aliquots. The results are shown in Table S-5.

Table S-5 LC-MS analysis of one aliquot of NJH2 after enrichment to 150 µL with the suggestion of the sum formula by Agilent Mass Hunter Qualitative B.07.00, the retention times (RT), the Δppm (<3 ppm) and the score (>90%).

Retention Times [min]	$[M+H]^+$	Formula	Score [%]	Δppm
11.31	164.0931	$C_7H_9N_5$	93.1	+0.62
19.40	199.0577	$C_5H_6N_6O_3$	99.2	-2.77
15.57	245.0995	$C_7H_{12}N_6O_4$	99.2	-1.78
17.22	289.1258	$C_9H_{16}N_6O_5$	99.2	-1.64
31.64	347.1464	$C_{15}H_{18}N_6O_4$	98.4	-0.83

G Semi-quantitative analysis of aldehydes in liquid inclusions

The quantification was done with the standard addition procedure. One quartz sample was added with the aldehydes decanal, dodecanal, tridecanal, tetradecanal, pentadecanal and hexadecanal in three concentrations (three replicates):

Decanal		[mg/L]		
	0.Addition	1. Addition	2. Addition	3. Addition
	[Area]	[Area]	[Area]	[Area]
	0	0.33	0.67	1
1M	942	15772	25261	41431
2M	1149	15696	27774	44569
3M	2513	18375	26340	43041
mean value	1535	16614	26458	43014
standard deviation	854	1525	1261	1569

	0	0.33	0.67	1
1M	8506	20478	32823	53250
2M	8275	22688	32440	53333
3M	7768	22412	31536	49961
mean value	8183	21859	32266	52181
standard deviation	378	1204	661	1923

Tridecanal		[mg/L]		
	0	0.33	0.67	1
1M	7599	14104	30427	47102
2M	6341	17337	30529	48503
3M	6656	17763	38650	47221
mean value	6865	16401	33202	47609
standard deviation	655	2001	4718	777

Tetradecanal		[mg/L]		
	0	0.33	0.67	1
1M	8089	23794	35799	48748
2M	8214	24109	36126	53650
3M	8663	25938	34587	48352
mean value	8322	24614	35504	50250
standard deviation	302	1158	811	2951

Hexadecanal		[mg/L]		
	0	0.33	0.67	1
1M	3574	17832	26520	48145
2M	2064	17586	25937	44889
3M	2594	16551	28736	44787
mean value	2744	17323	27064	45940
standard deviation	766	680	1477	1910

Pentadecanal		[mg/L]		
	0	0.33	0.67	1
1M	22363	32675		58206
2M	24189	32139	46407	60581
3M	27089	32729	45131	59946
mean value	24547	32514	45769	59578
standard deviation	2383	326	902	1230

	R^2	Regression funct. y=	[mg/L]	SD
Decanal	0.9905	40181x+1814.7	0.045	0.0210
Dodecanal	0.9816	42611x+7317.1	0.171	0.0089
Tridecanal	0.9899	41642x+5198.4	0.125	0.0157
Tetradecanal	0.994	40902x+9221.2	0.225	0.0074
Pentadecanal	0.9868	35440x+22882	0.646	0.0672
Hexadecanal	0.9842	41689x+2423.4	0.058	0.0184

The regression lines are displayed in the following plots:

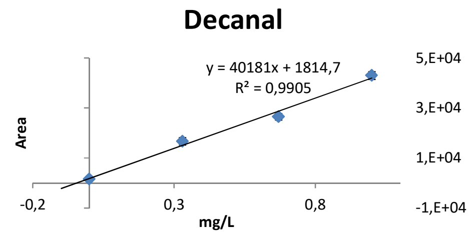

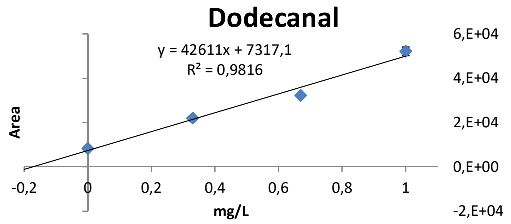

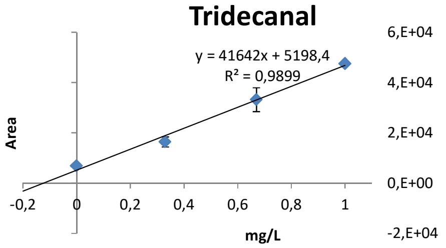

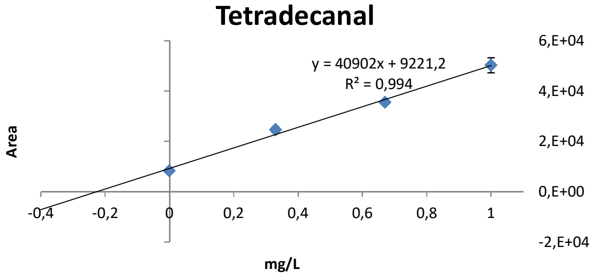

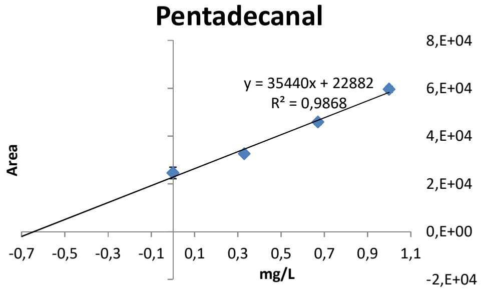

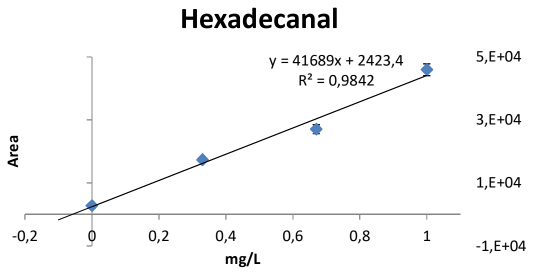

Based on these data, the concentration of each unspiked sample was calculated from the corresponding regression curve and listed in Table S-6:

Table S-6 Concentration of aldehydes in liquid inclusions

	[µg/kg]
Decanal	4.2 ± 2.0
Dodecanal	16.1 ± 0.9
Tridecanal	11.8 ± 1.5
Tetradecanal	21.2 ± 0.7
Pentadecanal	32.6 ± 6.3
Hexadecanal	5.5 ± 1.7

H Analysis of an olivine sample with GCxGC-MS

The GCxGC-MS analysis of an olivine from a mantle xenolith from a maar tuff ring shows only a few spots, which are also detected in the blanks.

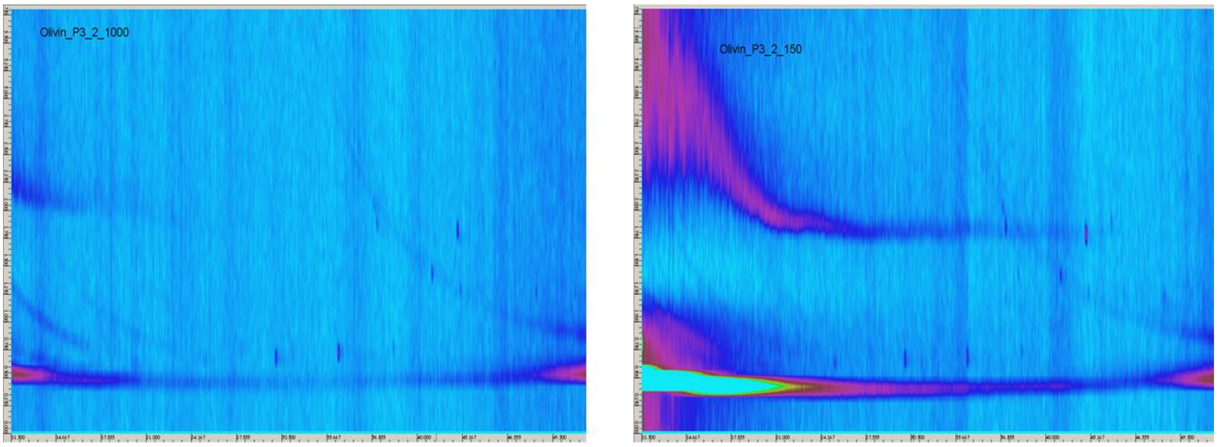

Figure S-10 GCxGC-MS analysis of one aliquot of olivine sample after enrichment to 1000 and 150 µL, respectively.

I References

1 Blewett, R.S., Czarnota, K., Henson, P.A., *Precambrian Res.* **183**, 2:203–229 (2010).

2 Kohn, B.P., Gleadow, A.J.W., Brown, R.W., Gallagher, K., Sullivan, P.B.O., Foster, D.A., *Austr. J. Earth Sci.* **49**, 4:697–717 (2002).

3 Cavosie, A.J., Wilde, S.A., Liu, D., Weiblen, P.W., Valley, J.W., *Precambrian Res.* **135,** 251–279 (2004).

4 Baxter, J.L., Wilde, S.A., Pidgeon, R.T., Fletcher, I.R., Geol. Conv., Macquarie University, North Ryde, 56–57 (abstract) (1984). The Jack Hills metasedimentary belt: an extension of the Early Archean Terrane in the Yilgarn Block, Western Australia. Australian

5 Elias, M., Geological Survey of Western Australia, Perth, WA, 1–22 (1983). Explanatory notes of the Belele geological sheet.

6 Harrison, T.M., Schmitt, A.K., McCulloch, M.T., Lovera, O.M., *Earth Planet Sci. Lett.* **268,** 476–486 (2008).

7 Wilde, S.A., Valley, J.W., Peck, W.H., Graham, C.M., *Nature* **409**, 175–178 (2001).

8 Occhipinti, S.A., Sheppard, S., Swager, C.P., Myers, J.S., Tyler, I.M., Sheet SG 50-7, Robinson Range, W.A. (2002).

9 Myers, J.S., Sheet SG 50-10, Byro, W.A. (1997).

10 Roedder, E. Fluid Inclusions, *Rev. in Mineralogy* 12, Mineralogical Society of America (1984).

11 Ueno, Y., Yamada, K., Yoshida, N., Maruyama, S., Isozaki, Y. *Nature* **440**, 516–519 (2006).

12 Shaw, C.S.J., Eyzaguirre, J., Fryer, B., Gagnon, J., *J. of Petrology* **46**, 5:945–972 (2005).

13 Mulder, I., Huber, S.G., Krause, T., Zetzsch, C., Kotte, K., Dultz, S., Schöler, H.F., *Chem. Geol.* **358**, 148-155 (2013).
